# Circulating fibroblast growth factor 23 and physical performance in middle-aged and older adults with normal kidney function

**DOI:** 10.1038/s41598-025-17367-6

**Published:** 2025-10-02

**Authors:** Keisei Kosaki, Shoya Mori, Masahiro Matsui, Chie Saito, Makoto Kuro-o, Kunihiro Yamagata, Seiji Maeda

**Affiliations:** 1 Institute of Health and Sport Sciences, University of Tsukuba, 1-1-1 Tennodai, Tsukuba, Ibaraki 305-8574 Japan; 2 Advanced Research Initiative for Human High Performance, University of Tsukuba, Tsukuba, Ibaraki Japan; 3 Division of Preventive and Sports Nephrology, Graduate School of Comprehensive Human Sciences, University of Tsukuba, Tsukuba, Ibaraki Japan; 4 Department of Nutritional Science, Faculty of Applied Bioscience, Tokyo University of Agriculture, Tokyo, Japan; 5 Department of Cardiology, Institute of Medicine, University of Tsukuba, Tsukuba, Ibaraki Japan; 6 Department of Child Psychiatry, Yokohama City University Hospital, Yokohama, Kanagawa Japan; 7 Department of Nephrology, Institute of Medicine, University of Tsukuba, Tsukuba, Ibaraki Japan; 8 Division of Mineral Metabolism, Center for Molecular Medicine, Jichi Medical University, Tochigi, Japan; 9 Faculty of Sport Sciences, Waseda University, Saitama, Japan

**Keywords:** Aging, Cardiorenal syndrome, Modifiable factor, Aerobic exercise capacity, Muscle strength, Ageing, Lifestyle modification, Preventive medicine

## Abstract

The circulating fibroblast growth factor 23 (FGF23) is a potential therapeutic target for cardiorenal syndrome. However, current evidence on the determinants, particularly the modifiable factors of circulating FGF23 levels that increase independently of the kidney function, remains limited. In this study, we aimed to investigate the association between physical performance measures and circulating FGF23 levels in middle-aged and older adults with normal kidney function. This cross-sectional study assessed circulating FGF23 levels and physical performance parameters, including the handgrip strength, knee extension strength, maximal gait speed, the 30-second chair stand test (30s-CST), sit-and-reach test, and aerobic exercise capacity in 158 participants. Multiple regression analyses were performed to evaluate the independent associations between circulating FGF23 levels and physical performance measures after adjusting for potential confounders including age, sex, the presence of lifestyle-related disease, serum phosphate and phosphate-regulating hormone, estimated glomerular filtration rate, and urinary albumin-to-creatinine ratio. Higher circulating FGF23 levels were associated with lower handgrip strength, knee extension strength, maximal gait speed, 30s-CST score, and aerobic exercise capacity. These associations remained significant after adjusting for confounders, except for the association with handgrip strength and aerobic exercise capacity, which was attenuated when renal function variables were included. However, when all physical performance parameters were included in a model, knee extension strength and aerobic exercise capacity were identified as independent determinants of circulating FGF23 levels. Physical performance, particularly knee extension strength, and to a lesser extent aerobic exercise capacity, was independently or partially associated with circulating FGF23 levels in individuals with normal kidney function. Maintaining physical performance may help regulate circulating FGF23 levels, highlighting a potential role in preventing its elevation.

## Introduction

Fibroblast growth factor-23 (FGF23) is an endocrine hormone secreted by osteocytes and osteoblasts that binds to the binary complex of fibroblast growth factor receptor and αKlotho expressed in renal tubular cells^1^. FGF23 inhibits phosphate reabsorption in the proximal tubules, thereby increasing phosphate excretion per nephron and contributing to increased urinary phosphate excretion. Thus, FGF23 is considered a phosphaturic hormone essential for the maintenance of phosphate homeostasis^2^. In contrast, a recent basic study demonstrated that elevated FGF23 levels can increase the risk of calcium-phosphate microcrystal formation in tubular fluid and induce renal tubular damage and interstitial fibrosis^3^. Furthermore, prospective cohort data have shown that individuals with higher circulating levels of FGF23 have a higher cumulative incidence of renal events (initiation of chronic dialysis or serum creatinine doubling) within 5 years, independent of serum creatinine levels^3^.

Growing evidence has increasingly supported a causal relationship between circulating FGF23 levels and worse cardiovascular outcomes as well as increased mortality^4^. Elevated circulating concentrations of FGF23 have been implicated as a potential contributor to the development of congestive heart failure, left ventricular hypertrophy, atrial fibrillation, and hypertension, regardless of the presence or absence of impaired kidney function^5–8^. A basic study also suggested that FGF23 binds to fibroblast growth factor receptor 4 on myocardial cells, leading to myocardial hypertrophy and potentially contributing to the pathogenesis of heart failure with preserved ejection fraction (HFpEF)^9^. These findings suggest that circulating FGF23 may not merely be an elevated surrogate marker resulting from renal dysfunction but also a mediator and potential therapeutic target of cardiorenal syndrome^10^. Consequently, the importance of preventing elevated circulating FGF23 levels is beginning to be recognized in clinical settings.

Circulating FGF23 levels increase progressively as kidney function declines, which is thought to be an adaptation to compensate for reduced nephron number and maintain phosphate homeostasis by increasing phosphate excretion per nephron^11^. In addition to impaired renal function, several factors that elevate circulating FGF23 levels have been identified, including parathyroid hormone,1,25-dihydroxyvitamin D (1,25(OH)_2_D), iron deficiency, inflammation, hypoxia, and calciprotein particles (colloidal nanoparticles composed of solid-phase calcium-phosphate and serum protein fetuin-A)^12,13^. However, current evidence regarding the determinants, particularly the modifiable factors, of circulating FGF23 levels that increase independently of kidney dysfunction, remains limited. Understanding these modifiable factors may contribute to the prevention of FGF23-induced cardiorenal syndrome.

Clinical evidence indicates that physical exercise may reduce circulating FGF23 levels. In patients with HFpEF, circulating FGF23 levels is independently associated with aerobic exercise capacity^14,15^. Furthermore, studies involving the oldest-old population reported circulating FGF23 levels are associated with several indicators of neuromuscular performance, including the muscle strength, balance, and aerobic exercise capacity^16,17^. In addition to these observational data, 6 months of resistance exercise, with or without blood flow restriction training, reportedly reduces circulating FGF23 levels in patients with stage 2 chronic kidney disease^18^. Although emerging evidence expands our understanding of the relationship between physical exercise and FGF23 levels, the association between circulating FGF23 levels and various physical performance metrics in relatively healthy middle-aged and older adults remains poorly understood. Clarifying this association may provide new insights for FGF23 management in clinical settings.

In this study, we aimed to investigate whether physical performance is associated with circulating FGF23 levels, independently of the kidney dysfunction. To achieve this, we focused on middle-aged and older adults with normal kidney function and examined the cross-sectional relationships between circulating FGF23 levels and objective measures of physical performance. We hypothesized that lower physical performance would be associated with higher circulating FGF23 levels among individuals with preserved renal function.

## Materials and methods

### Participants

This cross-sectional study used data from the baseline testing of our longitudinal study, The Aging Kidney Study, performed at the University of Tsukuba. This study has been registered with the University Hospital Medical Information Network Clinical Trials Registry (UMIN000034741). Of the individuals who participated in the baseline assessments, 184 participants were included in this study based on the following eligibility criteria: (1) age ≥ 45 years, and (2) estimated glomerular filtration rate (eGFR) ≥ 60 mL/min/1.73 m^2^ and urinary albumin-to-creatinine ratio (ACR) < 30 mg/g. This study was approved by the Ethics Committee of the University of Tsukuba Hospital (approval no. H30-161) and was performed according to the guidelines of the Declaration of Helsinki. Written informed consent was obtained from all the participants for study participation.

### Physical performances

The objective physical performance measures included handgrip strength, knee extension strength, maximal gait speed, 30-second chair stand test (30s-CST), sit-and-reach test (SRT), and submaximal exercise testing outcomes. Handgrip strength was measured using a Smedley-type dynamometer (T.K.K.5401; Takei Scientific Instruments, Niigata, Japan). Each participant performed two trials with each hand, alternating sides, and the average of the two highest values (one from each hand) was used for analysis^19^. The isometric knee extension strength was assessed in a seated position with approximately 90° flexion at both the hip and knee joints, using a validated handheld dynamometer (µTas F-1; ANIMA, Tokyo, Japan) following standardized procedures^20^. Participants were instructed to gradually exert maximal force and maintain full knee extension for approximately 3 s. Peak force during this period was recorded. Each leg was tested twice, and the average maximum values of both legs were used in the analysis. The handgrip strength and knee extension strength values were normalized to body weight, as it is strongly influenced by body weight. Maximal gait speed was determined by measuring the time (in seconds) needed by the participants to walk a 10-meter distance at their fastest possible pace^21^. The 30s-CST was conducted using a chair with a height of 40 cm without armrests, and the number of full stands completed within 30 s was recorded^22^. The SRT was conducted using a digital flexibility testing device (T.K.K.5412; Takei Scientific Instruments, Niigata, Japan). The participants were instructed to sit on the floor with their legs fully extended, maintaining contact between the wall and their hips, back, and occiput, with their arms extended forward and their elbows straight. From this position, they were instructed to slowly reach forward without bending their elbows, and the farthest point of reach was recorded. The SRT was performed twice, and the better of the two trials was adopted as the representative value^23^. A submaximal exercise test was conducted to assess the aerobic exercise capacity. The participants began exercising on a cycle ergometer at a workload of 20 W for 2 min, and continued exercising with incremental loading until they reached ventilatory threshold (VT), or up to 85% of their maximum heart rate (HR_max_) calculated using the age-predicted HR_max_ equation (i.e., 220 − age)^24,25^. While exercising, the workload was increased by 10–20 W per minute, and oxygen uptake and carbon dioxide output were continuously measured using an online computer-assisted circuit spirometry system (AE300S; Minato Medical Science, Osaka, Japan) to determine the VT. The VT point was determined using the V-slope method as the point at which carbon dioxide output increased exponentially relative to the oxygen uptake^26^.

### Circulating fibroblast growth factor 23

Circulating FGF23 concentrations were measured using serum samples and a commercial enzyme-linked immunosorbent assay kit (Kainos Laboratories, Inc., Tokyo, Japan)^27^. The intra- and inter-assay coefficients of variation were 2.8% and 2.6%, respectively, with a minimum detection level of 3.0 pg/mL. Blood samples were collected from the antecubital vein in the morning after overnight fasting, placed into serum separator tubes, centrifuged at 3,000 rpm for 15 min at 4 °C, and stored at − 80 °C until assay.

### Covariates

Individual lifestyle-related disease status was determined based on standard reference values and use of relevant medications. The criteria for each condition were as follows. Hypertension was defined as systolic blood pressure ≥ 140 mmHg, diastolic blood pressure ≥ 90 mmHg, or the use of antihypertensive medications. Dyslipidemia was identified by high-density lipoprotein cholesterol level < 40 mg/dL, low-density lipoprotein cholesterol level ≥ 140 mg/dL, triglyceride level ≥ 150 mg/dL, or the use of lipid-lowering medications. Diabetes mellitus was defined as a fasting blood glucose level ≥ 126 mg/dL, HbA1c level ≥ 6.5%, or use of glucose-lowering medications. Obesity/overweight was determined by body mass index ≥ 25 kg m^2^. Serum phosphate concentrations and hormones involved in phosphate metabolism, particularly 1,25(OH)_2_D and intact parathyroid hormone (int-PTH), were considered potential confounding factors. Serum 1,25(OH)_2_D and int-PTH levels were measured using a radioimmunoassay (Immunodiagnostic Systems Holdings Ltd., Boldon, United Kingdom) and electrochemiluminescence immunoassay (Roche Diagnostics K.K., Tokyo, Japan), respectively. Daily physical activity was assessed using a tri-axial accelerometer (Active Style Pro HJA-750 C; Omron Healthcare, Kyoto, Japan) worn on the left hip for 14 consecutive days, except during bathing or water-based activities. Valid data were defined as wearing the device for at least 10 h per day on a minimum of three days, including at least one weekend day^20^. The device estimated physical activity intensity in metabolic equivalents based on a validated algorithm. For this study, we focused on moderate-to-vigorous physical activity, defined as ≥ 3.0 metabolic equivalents.

### Statistical analysis

Data were reported as mean ± standard deviation or median [interquartile range] for continuous variables and frequency (%) for categorical variables. Circulating FGF23 levels exhibited a skewed distribution and were therefore log-transformed before analysis. Each physical performance parameter was categorized into two groups (i.e., higher or lower) based on its median value. The log-transformed circulating FGF23 concentrations between the higher and lower physical performance groups were compared using an independent *t* test. Hierarchical multiple linear regression analysis was conducted to examine the association between physical performance parameters and log-transformed circulating FGF23 levels, with adjustment for potential covariates.

The following hierarchical multiple linear regression models were established: Model 1 was the crude model; Model 2 was adjusted for age and sex; and Models 3 and 4 were additionally adjusted for lifestyle-related disease status (i.e., the presence of obesity/overweight, hypertension, dyslipidemia, and diabetes), serum phosphate and phosphate-regulating hormone (1,25(OH)_2_D and int-PTH) levels, eGFR, and urinary ACR. Furthermore, a stepwise linear regression analysis was conducted to determine the physical performance that was most strongly associated with circulating FGF23 levels; this analysis included all physical performance parameters along with potential covariates as independent variables. In the hierarchical and stepwise multiple linear regression analyses, all physical performance variables were treated as continuous variables. Statistical significance was set a priori at a *P* < 0.05. SPSS Statistics software (version 29.0; IBM Inc., NY, USA) was used for all statistical analyses.

## Results

Table 1 shows the characteristics of the study participants. After excluding patients with missing data on any of the covariates of interest (*n* = 23), who had undergone a nephrectomy in the past (*n* = 2), or who were not compliant with the study protocol (*n* = 1), 158 middle-aged and older adults (35 men and 123 women) with normal kidney function were included in the final analysis. The age of the participants ranged from 48 to 81 years. The mean circulating FGF23 concentrations (43.1 ± 16.5 pg/mL) and serum phosphate concentrations (3.48 ± 0.41 mg/dL) were within the normal range. None of the participants were taking medications related to FGF23-associated hypophosphatemic rickets/osteomalacia (i.e., human anti-FGF23 monoclonal antibodies).

**Table 1:** Participant characteristics.

Variables	Overall (*n* = 158)			Men (*n* = 35)			Women (*n* = 123)		
Age, years	63 ± 8			66 ± 8			62 ± 8		
Height, cm	158.5 ± 7.8			168.3 ± 7.3			155.8 ± 5.4		
Weight, kg	55.6 ± 9.8			64.7 ± 8.1			53.0 ± 8.7		
Body mass index, kg/m^2^	22.0 ± 3.0			22.8 ± 2.5			21.8 ± 3.1		
Overweight/obese, n (%)	24 (15)			5 (14)			19 (15)		
Hypertension, n (%)	30 (19)			11 (31)			19 (15)		
Dyslipidemia, n (%)	79 (50)			15 (43)			64 (52)		
Diabetes, n (%)	8 (5)			2 (6)			6 (5)		
Serum FGF23, pg/mL	40.5 [33.0–50.8]			43.8 [35.6–55.2]			39.8 [32.1–48.7]		
Serum 1,25(OH)_2_D, pg/mL	65.2 ± 17.2			67.3 ± 21.0			64.6 ± 16.0		
Serum int-PTH, pg/mL	38.0 [33.0–47.3]			38.0 [29.0–50.0]			38.0 [33.0–47.0]		
Serum phosphate, mg/dL	3.48 ± 0.41			3.19 ± 0.45			3.56 ± 0.35		
eGFR, mL/min/1.73m^2^	90.3 ± 15.1			83.4 ± 10.9			92.3 ± 15.6		
Urinary ACR, mg/g	9.0 [6.4–14.9]			5.9 [4.2–11.5]			9.6 [7.4–16.2]		
Handgrip strength, kgf/kg	0.50 ± 0.11			0.59 ± 0.10			0.47 ± 0.11		
Knee extension strength, kgf/kg	0.59 ± 0.17			0.64 ± 0.14			0.58 ± 0.17		
Maximal gait speed, m/sec	2.34 [2.12–2.66]			2.58 [2.30–2.96]			2.29 [2.07–2.58]		
30s-CST score, counts	20.6 ± 5.2			21.5 ± 5.0			20.4 ± 5.3		
SRT score, cm	36.4 ± 8.8			33.3 ± 8.9			37.3 ± 8.6		
$\dot{\text{V}}$O_2 VT_, mL/min/kg	14.3 [12.8–16.2]			15.3 [13.6–17.0]			14.1 [12.6–15.7]		
Daily physical activity, min/day	59.1 ± 24.2			54.2 ± 21.7			60.5 ± 24.8		

Values are presented as the means ± standard deviation or frequency counts (%). FGF23, fibroblast growth factor-23; 1,25(OH)_2_D, 1,25-dihydroxyvitamin D; int-PTH, intact-parathyroid hormone; eGFR, estimated glomerular filtration rate; ACR, albumin-to-creatine ratio; 30s-CST, 30-second chair stand test; SRT, sit-and-reach test; $\dot{\text{V}}$O_2 VT_, oxygen consumption at the ventilatory threshold.

**Fig. 1:**
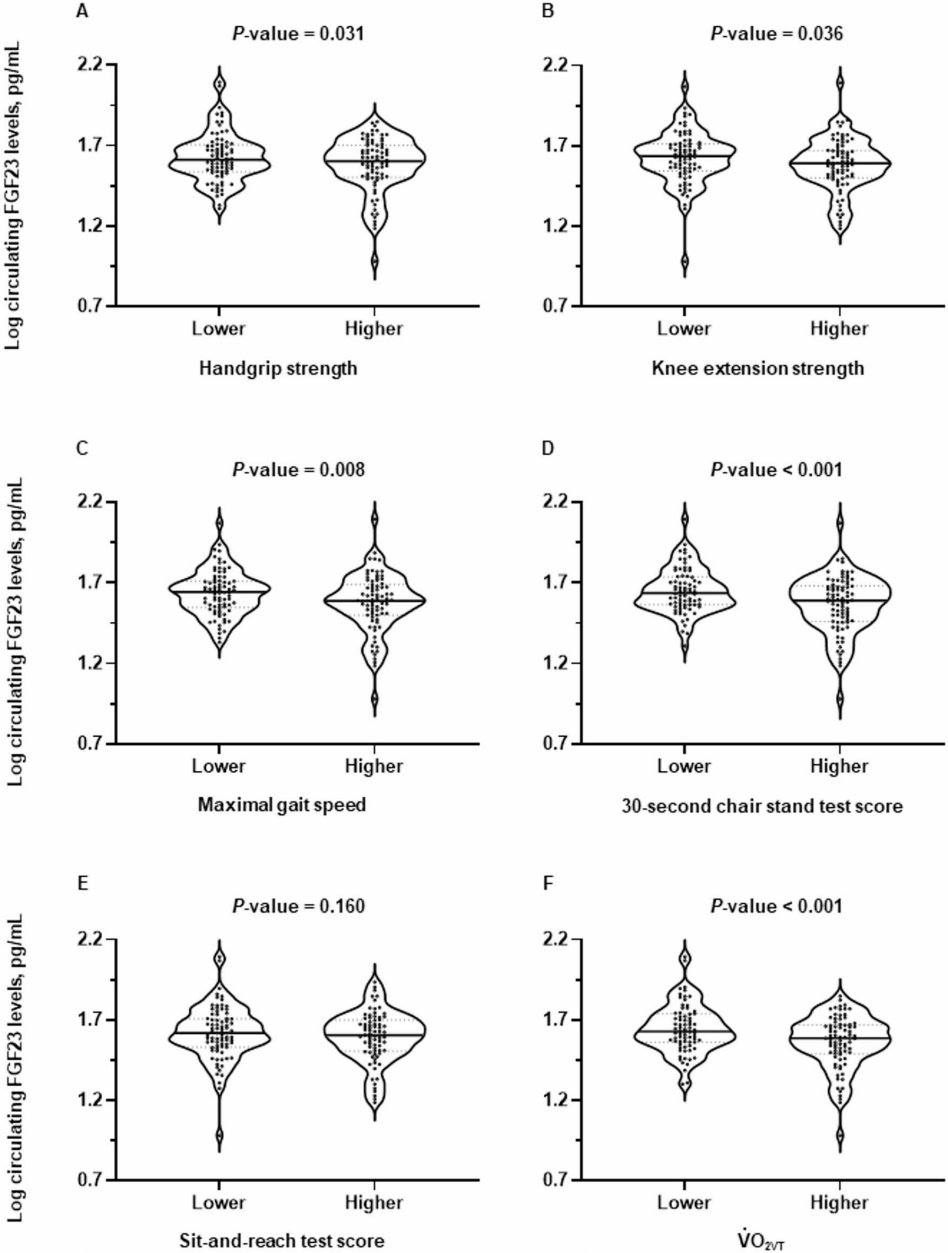
Differences in log-transformed circulating fibroblast growth factor 23 (FGF23) concentrations between two groups dichotomized according to the median values of handgrip strength (**A**), knee extension strength (**B**), maximal gait speed (**C**), the 30-second chair stand test score (**D**), the sit-and-reach test score (**E**), and oxygen consumption at ventilatory threshold ($\dot{\text{V}}$˙O_2VT_) (**F**).

Figure 1 presents a comparison of log-transformed circulating FGF23 concentrations between the higher and lower physical performance groups. Statistically significant differences were observed between the two groups in terms of the handgrip strength, knee extension strength, maximal gait speed, 30s-CST score, and aerobic exercise capacity. However, no significant between-group differences were observed in SRT score; comparison using circulating FGF23 concentrations before log transformation yielded similar results.

Table 2 shows the results of the hierarchical multiple linear regression analysis, with log-transformed circulating FGF23 levels as the dependent variable. In the crude model (Model 1), which included only physical performance parameters, handgrip strength, knee extension strength, maximal gait speed, 30s-CST score, and aerobic exercise capacity were significantly associated with circulating FGF23 levels. These associations remained statistically significant in Model 2, which was adjusted for age and sex, and in Models 3 and 4, which was additionally adjusted for lifestyle-related disease status, serum phosphate, phosphate-regulating hormone (1,25(OH)_2_D and int-PTH) levels, eGFR, and urinary ACR. However, the association of handgrip strength and aerobic exercise capacity with circulating FGF23 levels was attenuated in Model 4, which was adjusted for eGFR and urinary ACR in addition to potential confounding factors.

**Table 2:** Hierarchical multiple linear regression analysis of log-transformed Circulating FGF23 levels.

Variables	Model 1		Model 2		Model 3		Model 4	
	β	*P*-value	β	*P*-value	β	*P*-value	β	*P*-value
Handgrip strength	− 0.210	0.008	− 0.290	0.001	−0.176	0.088	−0.196	0.052
Knee extension strength	−0.238	0.003	−0.242	0.002	−0.171	0.037	−0.214	0.007
Maximal gait speed	−0.173	0.029	−0.177	0.037	−0.187	0.026	−0.243	0.003
30s-CST score	−0.228	0.004	−0.218	0.006	−0.186	0.020	−0.205	0.008
SRT score	−0.118	0.141	−0.103	0.197	−0.059	0.457	−0.080	0.304
$\dot{\text{V}}$O_2VT_	−0.275	0.001	−0.290	0.001	−0.230	0.021	−0.174	0.081

β indicates standardized regression coefficients. FGF23, fibroblast growth factor-23; 30s-CST, 30-second chair stand test; SRT, sit-and-reach test; $\dot{\text{V}}$O_2 VT_, oxygen consumption at the ventilatory threshold. Model 1 includes only each physical performance parameter (crude model). Model 2 is adjusted for age and sex. Model 3 is adjusted for Model 2 + overweight/obese, hypertension, dyslipidemia, diabetes, serum 1,25-dihydroxyvitamin D, serum intact-parathyroid hormone, serum phosphate, and daily physical activity. Model 4 is adjusted for Model 3 + estimated glomerular filtration rate and urinary albumin-to-creatine ratio.

Table 3 presents the results of the stepwise multiple linear regression analysis with all physical performance parameters and confounding factors entered sequentially. In Model 1, which included all physical performance parameters simultaneously, the knee extension strength was significantly associated with circulating FGF23 levels. In Model 2—which included all physical performance parameters, age, and sex—and Model 3—which further incorporated lifestyle-related disease status, serum phosphate and phosphate-regulating hormone (1,25(OH)_2_D and int-PTH) levels, aerobic exercise capacity and knee extension strength remained significant determinants of circulating FGF23 levels. In Model 4, which additionally included eGFR and urinary ACR, knee extension strength, eGFR, and the presence of hypertension remained significantly associated with circulating FGF23 levels.

**Table 3:** Stepwise multiple linear regression analysis of log-transformed circulating FGF23 levels.

Variables	B ± SE			β	*P*-value
Model 1 (R^2^ = 0.107, *P* < 0.001)					
$\dot{\text{V}}$O_2VT_, mL/min/kg	− 0.012 ± 0.004			− 0.238	0.003
Knee extension strength, kgf/kg	− 0.173 ± 0.078			− 0.178	0.027
Model 2 (R^2^ = 0.145, *P* < 0.001)					
$\dot{\text{V}}$O_2VT_, mL/min/kg	− 0.014 ± 0.004			− 0.279	< 0.001
Sex (female)	− 0.078 ± 0.031			− 0.201	0.012
Knee extension strength, kgf/kg	− 0.192 ± 0.077			− 0.197	0.013
Model 3 (R^2^ = 0.173, *P*< 0.001)					
$\dot{\text{V}}$O_2VT_, mL/min/kg	− 0.014 ± 0.004			− 0.283	< 0.001
Sex (female)	− 0.082 ± 0.030			− 0.211	0.007
Knee extension strength, kgf/kg	− 0.180 ± 0.076			− 0.185	0.019
Serum 1,25(OH)_2_D, pg/mL	− 0.002 ± 0.001			− 0.169	0.027
Model 4 (R^2^ = 0.206, *P* < 0.001)					
eGFR, mL/min/1.73m^2^	− 0.004 ± 0.001			− 0.347	< 0.001
Knee extension strength, kgf/kg	− 0.243 ± 0.072			− 0.249	< 0.001
Hypertension (yes)	0.062 ± 0.031			0.151	0.043

B and β indicate unstandardized and standardized regression coefficients, respectively. FGF23, fibroblast growth factor-23; $\dot{\text{V}}$O_2 VT_, oxygen consumption at the ventilatory threshold; 1,25(OH)_2_D, 1,25-dihydroxyvitamin D; eGFR, estimated glomerular filtration rate. A stepwise procedure is used to enter the following variables: Model 1 includes only each physical performance parameter. Model 2 includes Model 1 + age and sex. Model 3 includes Model 2 + overweight/obese, hypertension, dyslipidemia, diabetes, serum 1,25(OH)_2_D, serum intact-parathyroid hormone, serum phosphate, serum phosphate, and daily physical activity. Model 4 includes Model 3 + eGFR and urinary albumin-to-creatine ratio.

## Discussion

The main findings of this study are as follows. First, when comparing the groups classified by the median values of each physical performance parameter, circulating FGF23 levels were significantly higher in the group with lower handgrip strength, knee extension strength, maximal gait speed, 30s-CST score, and aerobic exercise capacity. Second, the results of linear regression analysis indicated that handgrip strength, knee extension strength, maximal gait speed, 30s-CST score, and aerobic exercise capacity were significantly negatively associated with circulating FGF23 levels. Third, the association between each physical performance parameter and circulating FGF23 levels remained statistically significant even after adjusting for potential covariates. Finally, when the relationships between all physical performance parameters and circulating FGF23 levels were examined simultaneously, aerobic exercise capacity and knee extension strength were identified as independent determinants of circulating FGF23 levels. Taken together, physical performance, particularly knee extension strength, and to a lesser extent aerobic exercise capacity, may be modifiable factors affecting circulating FGF23 levels in middle-aged and older adults with normal kidney function. These findings provide new insights into the management of circulating FGF23 levels in clinical settings.

FGF23 is a phosphaturic hormone that increases phosphate excretion per nephron^2^and a decline in kidney function (i.e., a reduction in the number of nephrons) leads to a compensatory increase in circulating FGF23 levels^11^; therefore, kidney function is the most potent determinant of FGF23. Nevertheless, recent epidemiological evidence suggests that an increase in circulating FGF23 levels may contribute to the pathogenesis of cardiovascular outcomes, independent of the kidney function decline^5–8^. In cross-sectional study, we focused on middle-aged and older adults with normal kidney function and identified the non-renal determinants associated with elevated circulating FGF23 levels. Our findings demonstrated that, even after adjusting for traditional FGF23 regulators, such as serum phosphate and phosphate-regulating hormones, various physical performance parameters, including knee extension strength, maximum gait speed, 30s-CST score, and aerobic exercise capacity, were independently associated with circulating FGF23 levels. These findings suggest that maintaining higher physical performance may be helpful in the prevention of FGF23 elevation in middle-aged and older adults with normal kidney function.

The independent associations between circulating FGF23 levels and various physical performance parameters observed in this study can be interpreted from an alternative perspective. Recent observational studies in older women demonstrated that higher circulating FGF23 levels are associated with reduced physical performance, including lower handgrip strength, slower gait speed, and higher prevalence of frailty^16^. Similarly, observational studies in very old populations reported that individuals with higher circulating FGF23 levels exhibit decreased lower extremity muscle strength, impaired balance function, reduced aerobic exercise capacity, and an increased risk of falls^17^. Considering the findings of our present study and those of previous studies, the relationship between circulating FGF23 levels and physical performance appears to be bidirectional.

Recent review articles have also discussed the potential mechanisms by which FGF23 contributes to muscle protein degradation and reduces exercise tolerance, such as the suppression of vitamin D metabolism, disruption of phosphate homeostasis, and increased oxidative stress^28^. More specifically, accumulating evidence suggests that elevated FGF23 may interfere with skeletal muscle metabolism by disrupting mitochondrial function, lowering energy production, and increasing oxidative stress. It may also inhibit the PI3K/Akt signaling pathway, which is essential for muscle growth and glucose uptake. These disruptions can impair muscle repair, reduce endurance, and contribute to muscle loss. Additionally, FGF23 has also been implicated in modulating the immune environment through the expression of pro-inflammatory cytokines, which may further exacerbate catabolic muscle conditions. Taken together, these findings suggest that, even in middle-aged and older adults with relatively preserved kidney function, FGF23 may function beyond its role as a phosphate-regulating hormone and is potentially involved in multiple pathways contributing to muscle weakness, including insulin resistance, oxidative stress, and inflammatory responses. However, several aspects of the direct and indirect effects of elevated circulating FGF23 levels on skeletal muscle and metabolic pathways remain unclear. Further longitudinal and mechanistic studies are warranted to elucidate the directionality and biological underpinnings of this association.

In a study involving patients with HFpEF, higher circulating FGF23 levels were independently associated with lower aerobic exercise capacity, as reflected by peak $\dot{\text{V}}$˙O₂ and 6-minute walk test distance^14,15^. Our findings confirmed that middle-aged and older adults with lower aerobic exercise capacity exhibited higher circulating FGF23 levels. Although the observation that the two distinct populations yielded similar results provided critical evidence for proposing novel determinants of FGF23, it should be noted that both studies employed a cross-sectional design.

In this study, the multiple linear regression analyses indicated that the association between aerobic exercise capacity and circulating FGF23 levels was partially attenuated after incorporating eGFR and urinary ACR into the regression model, whereas the relationship with knee extension strength persisted even after adjusting for kidney function. Therefore, elevated circulating FGF23 levels, independent of renal impairment, may contribute to decreased lower-extremity muscle strength. In contrast, elevated circulating FGF23 levels may affect the aerobic exercise capacity through various metabolic factors or other mechanisms. As the causal relationship between aerobic exercise capacity and circulating FGF23 levels remains unclear, prospective studies are needed to determine whether changes in aerobic exercise capacity are associated with alterations in circulating FGF23 levels.

Recent basic research has indicated that cardiac hypertrophy (particularly left ventricular hypertrophy) can lead to an increase in FGF23 production originating from cardiomyocytes rather than from osteocytes, thereby elevating circulating FGF23 levels^29^. Thus, even in middle-aged and older adults with preserved kidney function, subclinical left ventricular hypertrophy may enhance cardiomyocyte-derived FGF23 production, potentially causing mild elevations in circulating FGF23 levels. Moreover, such an elevation of cardiomyocyte-derived circulating FGF23 resulting from cardiac hypertrophy may not only alter phosphate homeostasis but also increase the overall metabolic burden, as well as hypertension and hemodynamic abnormalities. Cardiac remodeling can possibly initiate an increase in circulating FGF23 levels, which in turn exacerbates the decline in physical performance, forming a vicious cycle.

One strength of this study is the comprehensive assessment of physical performance while accounting for potential confounders, allowing for a more robust investigation of the association between circulating FGF23 levels and physical performance. However, this study has some limitations. First, owing to its cross-sectional design, it was difficult to infer causal relationships. Second, the study population consisted predominantly of middle-aged and older women with a relatively small proportion of men, suggesting a potential selection bias. Finally, because this was a small-scale observational study conducted at a single institution, caution should be exercised when considering the external validity of the findings. Future longitudinal and interventional studies are needed to track changes in circulating FGF23 levels and physical performance over time, and to determine whether maintaining or improving physical performance contributes to the regulation of circulating FGF23 levels. Moreover, the specific mechanistic pathways that could explain the independent association between circulating FGF23 levels and physical performance parameters remain to be elucidated in future experimental research.

In conclusion, this study demonstrated that various physical performance parameters were independently associated with circulating FGF23 levels in middle-aged and older adults with a normal kidney function. Among these parameters, knee extension strength and, to a lesser extent, aerobic exercise capacity remained significant determinants of circulating FGF23 levels even after adjusting for potential confounders. These findings suggest that maintaining physical performance may play a crucial role in preventing elevated circulating FGF23 levels in the absence of kidney impairment.

## Data Availability

The datasets generated and/or analyzed during the current study are not publicly available owing to ethical and legal constraints; however, anonymized data are available from the corresponding author upon reasonable request.
